# Intensive Case Management for Addiction to promote engagement with care of people with severe mental and substance use disorders: an observational study

**DOI:** 10.1186/s13011-017-0111-8

**Published:** 2017-05-25

**Authors:** Stéphane Morandi, Benedetta Silva, Philippe Golay, Charles Bonsack

**Affiliations:** Department of Psychiatry, Social Psychiatry Section, Community Psychiatry Service, Lausanne University Hospital (CHUV), Place Chauderon 18, 1003 Lausanne, Switzerland

**Keywords:** Assertive community treatment, Critical time intervention, Intensive Case Management, Addiction, Severe mental disorder, Substance use disorder, Engagement with care

## Abstract

**Background:**

Co-occurring severe mental and substance use disorders are associated with physical, psychological and social complications such as homelessness and unemployment. People with severe mental and substance use disorders are difficult to engage with care. The lack of treatment worsens their health and social conditions and increases treatment costs, as emergency department visits arise. Case management has proved to be effective in promoting engagement with care of people with severe mental and substance use disorders. However, this impact seemed mainly related to the case management model. The Intensive Case Management for Addiction (ICMA) aimed to improve engagement with care of people with severe mental and substance use disorders, insufficiently engaged with standard treatment. This innovative multidisciplinary mobile team programme combined Assertive Community Treatment and Critical Time Intervention methodologies. The aim of the study was to observe the impact of ICMA upon service use, treatment adherence and quality of support networks. Participants’ psychosocial and mental functioning, and substance use were also assessed throughout the intervention.

**Methods:**

The study was observational. Eligible participants were all the people entering the programme during the first year of implementation (April 2014–April 2015). Data were collected through structured questionnaires and medical charts. Assessments were conducted at baseline and at 12 months follow-up or at the end of the programme if completed earlier. McNemar-Bowker’s Test, General Linear Model repeated-measures analysis of variance and non-parametric Wilcoxon Signed Rank tests were used for the analysis.

**Results:**

A total of 30 participants took part in the study. Results showed a significant reduction in the number of participants visiting the general emergency department compared to baseline. A significantly decreased number of psychiatric emergency department visits was also registered. Moreover, at follow-up participants improved significantly their treatment adherence, clinical status, social functioning, and substance intake and frequency of use.

**Conclusions:**

These promising results highlight the efficacy of the ICMA. The intervention improved engagement with care and the psychosocial situation of people with severe mental and substance use disorders, with consequent direct impact on their substance misuse.

## Background

Only a minority of people with co-occurring severe mental and substance use disorders seek help and are treated for their problems [1–3]. On a personal level, important identified barriers to treatment are symptoms, lack of awareness of being in need of help, stigma or social problems such as homelessness or insufficient financial resources. On a structural level, the service location and organisation or the unavailability of addiction specialists have been recognised as care access limitations [4, 5]. Absence of care can lead to health and social complications and contributes to higher costs of public services as emergency department visits arise [6]. Different models of case management have proved to be effective in promoting engagement with care of people with substance use disorders in a variety of settings [7–9]. Case management also showed to reduce substance misuse among homeless people with severe mental illness [10]. However, the impact of these interventions seemed to be mainly determined by the case management reference model [11]. Assertive community treatment (ACT) improved housing stability and was cost-effective for homeless people with severe mental and substance use disorders, reducing inpatient and emergency department visits. Critical Time Intervention (CTI) showed promise for housing support, psychiatric symptoms and substance use in this population.

In 2001, an Intensive Case Management (ICM) programme for people with severe mental disorders (in French *Suivi Intensif dans le milieu*-SIM), was developed and tested in Lausanne, Switzerland, an urban area of 265′000 inhabitants [12]. The intervention combined the Assertive Community Treatment (ACT) [13, 14] and the Critical Time Intervention [15] methodologies. As in the ACT model, case managers and psychiatrists provided home visits when needed. A caseload limited to a maximum of 20 clients per full-time professional allowed case managers to spend more time with each person and to intensify the follow-up during crisis periods. The multidisciplinarity of the team granted an approach that was not exclusively focused on the illness. Each professional could discuss and provide specific help on a wider range of issues, such as housing or income. These specificities of ACT are key elements that contribute to clients’ satisfaction and promote their engagement with care [16]. The ICM programme in Lausanne differed from ACT in three aspects. First, because of a lack of resources, the team was only available between 8 a.m. and 6 p.m. During nights and weekends, clients could be referred to the local psychiatric emergency department (ED). Second, situations were regularly discussed among team members, but each client was followed by a specific case manager and by the psychiatrist when no other doctor was involved in the situation. Third, the team members only delivered services that other professionals could not provide, such as intensive home visits or practical help for time consuming administrative procedures. This led to a closer collaboration with other members of the health and social network and made discharge towards other services easier. The ICM programme borrowed also elements from the critical time intervention model (CTI) [15]: 1. The intervention was time-limited to critical or transitional periods; 2. It aimed to engage clients with other services through a smooth process; 3. During the programme, it offered a psychological as well as a practical help adapted to client’s needs; 4. Client’s resources and limitations were assessed in vivo and practical solutions proposed.

In Lausanne, the ICM intervention has proved to be effective in promoting engagement with care of people with severe mental illness and improving both their clinical and social functioning [17]. These results were in line with international studies on ICM for severe mental disorders that have shown to reduce hospitalisations, increase participants retention in care and improve their social functioning [18]. Based on the ICM model, in 2014 a pilot project of Intensive Case Management for Addiction (ICMA) (in French *Suivi Intensif dans le milieu pour les problèmes d’addiction* - SIMA) was developed and implemented in the same area. The programme was tested with a group of hard-to-reach people with severe mental and substance use disorders, who have difficulties to engage with addiction or psychiatric services. This paper presents findings from the ICMA observational study.

### Aims of the study

The main aim of the study was to test whether ICMA improved engagement with care of people with severe mental and substance use disorders. Specifically, expected primary outcomes were: decreased rates of unplanned service use and involuntary hospitalisations, improved level of treatment adherence and enhanced quality of primary (relatives) and secondary (caregivers) support networks. The secondary objective was to evaluate the programme impact on participants’ well-being through the measure of their social conditions (housing, legal status and criminal records), clinical status, social functioning, and alcohol and other illicit drug use.

## Methods

### Sample

The ICMA programme was addressed to people with severe mental and substance use disorders hard-to-reach or refusing traditional addiction or psychiatric treatment. ICMA participants repeatedly failed to attend outpatient appointments and/or were involuntarily hospitalised with no ambulatory care options after discharge. Eligible study participants were every consecutive person entering the programme during the first year of implementation between April 2014 and April 2015. Inclusion criteria for the programme were: to be aged between 18 and 65 years and to live in the urban area of Lausanne, Switzerland. Exclusion criterion for the programme was the participant’s ability to collaborate with an addiction treatment or the psychiatric services. Data were collected through structured questionnaires and medical charts. Assessments were conducted at baseline (T0) and at 12 months follow-up or at the end of the programme if completed earlier (T1).

During the first year of implementation, 30 participants entered the programme and were eligible for the study (Table 1). They were mainly male (73%), single (63%) and with an average age of 39 years. Half were Caucasian (50%) while 30% were mixed-race. Only 33% were native of Switzerland. Ninety-seven percent were unemployed, although 37% had achieved a secondary or higher education degree. The primary diagnosis was mental and behavioural disorder due to psychoactive substance use (57%), especially alcohol (59%), followed by schizophrenia, schizotypal and delusional disorders (20%), affective disorder (13%) and personality disorder (10%). Eighty-three percent of the participants were hospitalized at least once in their life, the average age of first admission was 31 years and 57% had at least one involuntary hospitalization. More detailed socio-demographic and clinical characteristics of the participants were published elsewhere [2].

**Table 1:** Baseline characteristics (*N* = 30)

Characteristics	
Demographics	
Age (mean ± SD)	38.90 ± 10.50
Sex, % Male (n)	73.3% (22)
Education % (n)	
None	23.3% (7)
Compulsory education	40.0% (12)
Secondary education	30.0% (9)
Tertiary education	6.7% (2)
Marital status % (n)	
Single	63.3% (19)
Married/Registered partnership	6.7% (2)
Other ^a^	30.0% (9)
Ethnicity % (n)	
Caucasian	50.0% (15)
African American	16.7% (5)
Asian	3.3% (1)
Other^b^	30% (9)
Origin	
% Born in Switzerland (n)	33.3% (10)
Employment status	
% Unemployed (n)	96.7% (29)
Clinical history % (n)	
Age of first admission (mean ± SD)	30.84 ± 10.32
Hospitalized at least once	83.3% (25)
Hospitalized at least once involuntary	56.7% (17)
Main diagnosis (ICD-10) % (n)	
Mental and behavioural disorders due to psychoactive substance use (F10-F19)	56.7% (17)
Schizophrenia, schizotypal and delusional disorders (F20-F29)	20.0% (6)
Mood [affective] disorders (F30-F39)	13.3% (4)
Disorders of adult personality and behaviour (F60-F69)	10.0% (3)

Note. ^a^ divorced/widowed/separated; ^b^ person of mixed race

### Intervention description

Two full-time case managers, one nurse and one social worker, and a 20% psychiatrist were recruited for the project. Participants were addressed to ICMA programme by their relatives, or by their health or social professionals. Programme admission and objectives were discussed during multidisciplinary team meetings. When needed, contacts were made with other professionals already involved in the situation. If inclusion criteria were met, the situation was assigned to a case manager. The referring relative or professional had to be present during the first contact with the participant in order to share their concerns and to explain why the intervention was requested. If the participant disagreed with the concerns of the referring relative or professional, they were encouraged to express their own expectations and needs. The intervention was then focused on the participant’s agenda. Most of the time, participants identified a social problem, such as finding a home or a source of income, as their main concern. This allowed the case managers or the psychiatrist to provide a practical support and to develop the therapeutic relationship. This practical help also gave the opportunity to follow the participant during their daily activities and to assess their resources and limitations in vivo. Based on these observations, the participant’s support network and the recovery plan were progressively developed.

The programme was completed when another addiction treatment or psychiatric service was permanently in charge of the participant. The decision to end the ICMA was always taken by the case manager in accordance with the participants and the other care providers. The intervention could also end if participants moved out of the catchment area, if they were lost to follow-up, if they refused to go on with the programme or in case of death.

### Measures

Socio-demographic characteristics, diagnoses and clinical history data were collected at baseline through structured questionnaires and medical charts. Primary and secondary outcomes measures were assessed at baseline (T0) and at 12 months follow-up or at the end of the programme if completed earlier (T1).

#### Primary outcomes

The primary outcome measures focused on service use, treatment adherence and quality of primary (relatives) and secondary (caregivers) support networks. Service use data before and during programme were provided by medical charts. Namely, the researchers assessed whether or not participants had been hospitalized (voluntary and/or involuntary) in a psychiatric or addiction treatment unit or had been admitted in the general or psychiatric ED at least once during the reference period. The frequency of readmission and contact with the ED, and the number of inpatient days were also recorded.

Treatment adherence was assessed by case managers on the basis of two items rating appointment and medication adherence on a visual-analogic scale ranging from 0 (no adherence) to 100 (total adherence). Two other treatment adherence items assessing psychotropic medication compliance and appointment attendance were incorporated in the Health of the Nation Outcome Scale (HoNOS) [19], which is routinely assessed by clinicians at the institutional level. The HoNOS evaluates mental and social functioning through 12 observer-rated items, quoted on a Likert – type scale from 0 (no problems during the reporting period) to 4 (severe to very severe problem during the reporting period). The French HoNOS has been shown to have moderate internal consistency, excellent test-retest reliability and good inter-rater reliability [20]. The predictive validity of HoNOS has always been modest and the French version is no exception. However it has been shown to be suitable for use at the item level for discriminating clinically meaningful clusters of patients [21].

The quality of primary and secondary support networks was evaluated by case managers through the Support Network Scale [22], with anchors ranging between 1) adequate and helpful, and 2) inadequate (gathering the answers: exhausted and overwhelmed, inactive and unstable, inadequate and incompetent, absent and nonexistent).

#### Secondary outcomes

Secondary outcome measures combined data on participants’ housing conditions (stable housing vs. homeless), legal status (legal guardianship, involuntary hospitalization and/or penal measures underway), criminal records (number of participants with at least one crime, infraction and/or victimisation occurred during the previous 12 months), psychosocial and mental functioning, and alcohol and other illicit drug use in the previous 30 days.

To assess participants’ psychosocial and mental functioning several validated and widely used scales were deployed. The 12 observer-rated items of the HoNOS [19] were assessed. Item-level scores rather than composite scores were used in the analysis [21]. The Crisis Triage Rating Scale (CTRS) assess participants’ dangerousness, ability to cooperate and support system on the basis of three Likert – type subscales ranging from 0 (no problems during the reporting period) to 4 (severe to very severe problem during the reporting period) [23, 24]. The subscales Ability to cooperate and Support system were also analysed as primary outcome measures of treatment adherence and quality of primary and secondary support networks. The CTRS has been validated in English, showing good reliability and validity [23, 24]. The French version has been shown to be sensible to change in assertive community treatment settings [17].

The Global Assessment of Functioning Scale (GAF) rates the participants’ social, occupational and psychological functioning on a numeric scale from 1 to 100 [25]. The Clinical Global Impression – Severity scale (CGI-S) evaluates illness severity at the time of assessment on a 7-point scale quoting from 1 (normal) to 7 (among the most extremely ill) [26]. The GAF (which is the DSM-IV fifth axis) and CGI-S are clinical global impression scales for which inter-rater reliability has been shown to be satisfactory to excellent [27, 28].

Alcohol and other illicit drug use in the previous 30 days were self-reported. A structured questionnaire was administered by case managers to assess whether or not participants had been using alcohol and/or other illicit drug at least once during the last month. Namely, the case managers aimed at assessing the alcohol and other illicit drug use frequency, the average number of alcohol units consumed per drinking day and if the participants had been part at least one time of a heavy alcohol use episode (> than 10 alcohol units).

### Statistical analysis

Primary and secondary dichotomous outcomes were analysed using McNemar-Bowker’s exact test. General Linear Model repeated-measures analysis of variance was performed for continuous and ordinal variables. Highly skewed continuous and ordinal variables were analysed using non-parametric Wilcoxon Signed Rank tests.

Baseline data were compared with 12 months follow-up measures or with final assessment if the programme was completed earlier.

In order to verify whether longer engagement in the programme impacted outcomes at T1, the relationship between programme duration and the outcomes’ variations (T0 vs. T1) was tested using Spearman’s rank correlation coefficient. These analyses revealed no impact of the programme duration on the outcomes.

Services use data, before and during programme, were compared over the same time span (i.e.: 6 months before vs. 6 months during programme; 8 months vs. 8 months; etc.) based on each individual programme length, but no longer than the 12 months evaluation point.

Assuming a sample size of 30 and interest in moderate sized effects (i.e., Cohen’s d ≥ 0.5; described as observable and noticeable to the eye of the beholder) and using a conservative estimate of the correlation between time 1 and time 2 measurements, 72% power for the comparison of pre- and post-measurements could be achieved adjusting for the use of the Wilcoxon test. Assuming even a reasonable correlation between the first and the second measurements (*r* = 0.7), the power becomes 90.8% which could be considered as more than adequate. Deviations from normality would further increase power.

All statistical tests were two-tailed and significance level was set at .05. Statistical analyses were performed with the IBM SPSS statistical package version 23.

## Results

Out of 30 participants enrolled at the baseline, 17 were still undergoing the programme after 12 months while 13 had completed it. At the end of ICMA intervention, 2 participants were transferred to other services of the Department of psychiatry, 2 to the alcohology service, 2 to private psychiatrists, 3 to other psychosocial services and 3 were not referred to any service (one improved sufficiently, one moved and the last one refused to be referred to another service). One 50 years old participant died during the programme. The cause of the death was undetermined. The mean programme duration was 10.00 ± 2.83 months. During the first year of implementation, each participant had on average 1.25 contacts per week with the case manager (1.07 h per week).

### Primary outcomes

No significant influence of the programme duration on the primary outcomes’ variations was found.

Longitudinal analysis comparing service use over the same time span before and during programme (Table 2) showed a significant decreased rate of general ED contacts (73% to 50%; *p* = .039). The decrease in the number of contacts with the psychiatric ED (Wilcoxon z = −1.997; *p* = .046; *r* = −.36) was also significant, with on average 0.60 ± 1.22 (Mdn = 0.0; IQR = 1) contacts before starting the programme and 0.20 ± 0.55 (Mdn = 0.0; IQR = 0) during the following period. No significant differences were found for the number of voluntary and involuntary psychiatric hospitalisations, the number of general ED visits and the number of total inpatient days. The decreased rate of involuntary hospitalizations (33% to 13%) did not reach statistical significance.

**Table 2:** Longitudinal analysis comparing services use over the same time span before and after programme enrolment

Services use	Before (*N* = 30)	After (*N* = 30)	Test	*p*-value
Psychiatric hospitalisation % (n)	63.3% (19)	53.3% (16)	^a^	.508
Involuntary hospitalisation % (n)	33.3% (10)	13.3% (4)	^a^	.070
Psychiatric ED visit % (n)	30.0% (9)	13.3% (4)	^a^	.125
General ED visit % (n)	73.3% (22)	50.0% (15)	^a^	.039
Number of psychiatric hospitalisations Mdn (IQR)	1.0 (2)	1.0 (2)	*z* = −0.354^b^	.723
Number of involuntary hospitalisations Mdn (IQR)	0.0 (1)	0.0 (0)	*z* = −1.327^b^	.185
Number of psychiatric ED visits Mdn (IQR)	0.0 (1)	0.0 (0)	*z* = −1.997^b^	.046
Number of general ED visits Mdn (IQR)	2.0 (3)	0.5 (2)	*z* = −1.573^b^	.116
Number of inpatient days Mdn (IQR)	22.0 (45)	6.5 (49)	*z* = −0.503^b^	.615

Note. ^a^ McNemar-Bowker’s Test; ^b^ Wilcoxon Signed Rank test; *Mdn* median, *IQR* interquartile range

Participants’ treatment adherence improved significantly during the programme (Table 3). At T1, participants scored significantly better on medication adherence (F(1,23) = 15.754, *p* = .001, ƞ_p_^2^ = .407) and appointment adherence (F(1,29) = 9.604, *p* = .004, ƞ_p_^2^ = .249). Besides, the severity scores on the two additional HoNOS-based items testing participants’ appointment attendance (F(1,27) = 12.911, *p* = .001, ƞ_p_^2^ = .323) and psychotropic medication compliance (F(1,23) = 10.827, *p* = .003, ƞ_p_^2^ = .320) decreased significantly. The enhanced participants’ compliance was also confirmed by the reduced score achieved at T1 on the CTRS Ability to Cooperate subscale (F(1,28) =16.605, *p* < .001, ƞ_p_^2^ = .372).

**Table 3:** Clinical and social within-group changes at 12 months follow-up or at the end of the programme if completed earlier (T1) compared to baseline (T0)

Outcome measures	T0 (*N* = 30)	T1 (*N* = 30)	*p*-value
Housing conditions			
% Homeless (n)	24.1% (7)	6.9% (2)	.125^a^
Legal status % (n)			
Legal guardianship underway	63.3% (19)	73.3% (22)	.375^a^
Involuntary hospitalization underway	30.0% (9)	23.3% (7)	.687^a^
Penal measure underway	13.3% (4)	16.7% (4)	1.000^a^
Criminal records during the previous 12 months (*N* = 17) % (n)			
Crime or infraction	47.1% (8)	35.3% (6)	.688^a^
Victimisation	35.3% (6)	5.9% (1)	.063^a^
Treatment adherence (mean ± SD) (Visual-analogic scales)			
Medication adherence	52.92 ± 32.36	78.75 ± 29.68	.001^b^
Appointments adherence	52.10 ± 34.22	74.50 ± 26.37	.004^b^
Network support scale % (n)			
Adequate and helpful	10.0% (3)	66.7% (20)	<.001^a^
Inadequate (exhausted,inactive, incompetent, absent)	90.0% (27)	33.3% (10)	
CTRS (mean ± SD)			
Dangerousness Subscale	2.79 ± 1.34	1.55 ± 1.29	<.001^b^
Support System subscale	2.75 ± 1.23	1.43 ± 1.34	.001^b^
Ability to cooperate subscale	2.69 ± 1.07	1.90 ± 1.01	<.001^b^
HoNOS items (mean ± SD)			
Overactive, aggressive, disruptive or agitated behaviour	1.28 ± 1.53	0.83 ± 1.25	.130^b^
Non-accidental self-injury	0.79 ± 1.17	0.31 ± 0.85	.041^b^
Problem drinking or drug-taking	3.55 ± 0.87	2.52 ± 1.12	<.001^b^
Cognitive problems	2.24 ± 1.45	1.66 ± 1.39	.009^b^
Physical illness or disability problems	1.00 ± 1.27	0.79 ± 1.31	.364^b^
Problems associated with hallucinations and delusions	1.15 ± 1.40	0.81 ± 1.23	.071^b^
Problems with depressed mood	2.86 ± 1.24	1.46 ± 1.29	<.001^b^
Other mental and behavioural problems	2.36 ± 1.68	1.72 ± 1.30	.115^b^
Problems with relationships	2.97 ± 0.86	2.14 ± 0.99	<.001^b^
Problems with activities of daily living	2.93 ± 1.25	2.38 ± 0.97	.011^b^
Problems with living conditions	2.45 ± 1.68	1.45 ± 1.50	.003^b^
Problems with occupation and activities	3.41 ± 0.68	2.48 ± 1.09	<.001^b^
Appointments attendance (additional item)	2.32 ± 1.16	1.43 ± 1.16	.001^b^
Psychotropic medications compliance (additional item)	2.21 ± 1.71	1.13 ± 1.07	.003^b^
Clinical Global Impression – Severity scale (CGI-S) (mean ± SD)	5.45 ± 1.15	4.59 ± 1.05	.002^b^
Global Assessment of Functioning Scale (mean ± SD)	28.10 ± 9.93	37.28 ± 8.92	<.001^b^

Note. ^a^ McNemar-Bowker’s Test; ^b^ General Linear Model repeated-measure

Finally, the support network quality improved significantly. Sixty-seven percent of the participants’ primary (relatives) and secondary (professionals) networks were described by case managers as “adequate and helpful” at T1 versus only 10% at baseline (*p* < .001). A significant improvement was also achieved on the CTRS Support System subscale (F(1,27) = 12.680, *p* = .001, ƞ_p_^2^ = .320).

### Secondary outcomes

Secondary outcomes are reported in Table 3. No significant influence of the programme duration on the secondary outcomes’ variations was found.

Participants’ psychosocial and mental functioning improved significantly during the programme. The item-level HoNOS analysis showed significant ameliorations at T1. After 12 months (or at the end of the programme if completed earlier), participants decreased their severity scores on eight items out of 12: Non-accidental self-injury (F(1,28) = 4.589, *p* = .041, ƞ_p_^2^ = .141), Problem drinking or drug-taking (F(1,28) = 24.852, *p* < .001, ƞ_p_^2^=.470), Cognitive problems (F(1,28)=7.965, *p* = .009, ƞ_p_^2^ = .221), Problems with depressed mood (F(1,27) = 27.842, *p* < .001, ƞ_p_^2^ = .508), Problems with relationships (F(1,28) = 18.453, *p* < .001, ƞ_p_^2^ = .397), Problems with activities of daily living (F(1,28) = 7.451, *p* = .011, ƞ_p_^2^ = .210), Problems with living conditions (F(1,28) =10.684, *p* = .003, ƞ_p_^2^ = .276), Problems with occupation and activities (F(1,28) = 20.786, *p* < .001, ƞ_p_^2^ = .426). While only 5 of these results remained significant after correction for multiple comparison (Problem drinking or drug-taking; Problems with depressed mood; Problems with relationships; Problems with living conditions; Problems with occupation and activities), the total number of significant differences was well above what could be expected by chance (12 × 0.05 = 0.6 comparison with *p*-values of .05 or lower).

A significant positive change was also registered on the CTRS Dangerousness Subscale (F(1,28) = 31.832, *p* < .001, ƞ_p_^2^ = .532). These results were further confirmed by the GAF and the CGI-S. Participants’ global functioning improved significantly during the programme (F(1,28) =24.207, *p* < .001, ƞ_p_^2^ = .464) while CGI-S scores decreased significantly (F(1,28)=11.290, *p*=.002, ƞ_p_^2^ = .287).

Analysis showed no significant changes concerning participants’ housing conditions and legal status. Nevertheless, only 7% of the participants were still homeless at T1 compared to the 24% at T0. The decreased trend of victimisations (35% to 6%) of participants between the 12 months before and after the beginning of the programme was not significant.

Self-reported alcohol and other illicit drug use results are reported in Table 4. Comparing T0 and T1, no significant differences were found concerning the number of alcohol consumers during the previous 30 days. Nevertheless, the average number of alcohol units consumed per drinking day decreased significantly, from 12.26 ± 11.61 at the baseline to 5.48 ± 8.53 at T1 (F(1,26) = 8.246, *p* = .008, ƞ_p_^2^ = .241). Similar results were found for the rate of heavy drinkers: while 71% of the participants had been part at least once of a heavy alcohol use episode (> than 10 alcohol units) at T0, only 32% reported a similar episode at T1 (*p* = .001). A significant result was also found at T1 for the frequency of alcohol use (Wilcoxon z = −2.721, *p* = .007).

**Table 4:** Alcohol and other illicit drug use within-group differences and 12 months follow-up or at the end of the programme if completed earlier (T1) compared to baseline (T0)

Outcome measures	T0 (*N* = 30)	T1 (*N* = 30)	Test	*p*-value
Alcohol use during the last 30 days				
% Consumers (n)	86.2% (25)	68.9% (20)	a	.125
Alcohol units per drinking day (mean ± SD)	12.26 ± 11.61	5.48 ± 8.53	F(1,26) = 8.246	.008
At least one episode of heavy alcohol use % (n)	71.4% (20)	32.1% (9)	a	.001
Frequency % (n)				
Almost every day	51.7% (15)	27.6% (8)	z = −2.721^b^	.007
3–4 days per week	10.3% (3)	6.9% (2)		
1–2 days per week	20.7% (6)	13.8% (4)		
1–3 days per month	3.5% (1)	20.7% (6)		
none	13.8% (4)	31.0% (9)		
Other illicit drug use during the last 30 days				
% Consumers (n)	68.9% (20)	44.8% (13)	a	.016
Frequency % (n)				
Almost every day	27.6% (8)	6.9%(2)	z = −3.064^b^	.002
3–4 days per week	3.5% (1)	6.9% (2)		
1–2 days per week	20.7% (6)	6.9% (2)		
1–3 days per month	17.2% (5)	24.1% (7)		
none	31.0% (9)	55.2% (16)		

Note. ^a^ McNemar-Bowker’s Test; ^b^ Wilcoxon Signed Rank test; *SD* standard deviation

The rate of other illicit drug use decreased significantly, from 69% at baseline to 45% at T1 (*p* = .016). Furthermore, the frequency of use at T1 was significantly reduced compared to the baseline (Wilcoxon z = −3.064, *p* = .002).

## Discussion

Throughout the intervention, ICMA significantly reduced the number of participants visiting the general ED and the number of psychiatric ED visits. Moreover, at follow-up participants showed significant improvements in their treatment adherence, clinical status, social functioning and the rates and frequency of alcohol and other illicit drug use. These findings were in line with previous studies on the effectiveness of ICM in population with severe mental and substance use disorders [18, 29]. From the local authorities’ point of view, they justified the high costs of the programme due to the small caseload, the high intensity of the intervention and the very specific target population. However, the long term programme cost-effectiveness should be assessed in the future.

The ICMA intervention was able to overcome barriers to care access on both the personal and the structural level. On the personal level, immediate practical help focused on participant’s needs allowed this latter to build a strong relationship with the care provider avoiding distrust and suspicion. The experience of a positive therapeutic relationship erased the memory of bad previous experiences with care services such as coercion, and overcame the addiction behaviours. On the provider’s level, mobility, proactivity, commitment and availability of care offered the opportunity to answer participants’ demands. Finally, on the system’s level, the intervention mobilized the network, eased the care coordination and allowed the development of new therapeutic options.

### Emergency Department use, hospitalisation, coercion, support network quality

Appointment attendance and medication compliance increased during the intervention. Participants’ improved treatment adherence significantly reduced the rate of admission to the general ED and the number of psychiatric ED visits. However, no significant impact was found on the number of people admitted to the psychiatric hospital. This could be explained by the heterogeneity of the sample. Before the intervention, some participants were regularly hospitalised or had had long inpatient stays. In these situations, the ICMA allowed a prompt discharge and return into the community. Other participants had had no contact with care services for long periods before the enrolment. Therefore, once in the programme, their highly decayed health status required immediate inpatient care. The small sample size did not allow subgroup analysis that could have had possibly show differences between “heavy service users” and participants who avoided care before entering the programme as highlighted by previous studies [30, 31]. If the rate and the number of psychiatric admissions did not decrease, the use of compulsion seemed positively, but not significantly, reduced.

The quality of the support network was greatly improved by the ICMA intervention. An important part of the case managers’ and the psychiatrist’s work was dedicated to develop and enhance the collaboration with other social and health services as well as participants’ relatives, and to improve care coordination. On a system level, regular appointments with other services’ management staff were organised. Moreover, case managers weekly attended other services team meetings. On a clinical level, regular contacts with each member of the network were constantly fostered.

### Clinical and social outcomes and substance use

Most clinical and social outcomes improved during the intervention. If the social functioning improvements can easily be explained by the fact that case managers and the psychiatrist provided practical help and supported the participants during social procedures, their clinical evolution needs to be further discussed. Clinical improvements may be explained by three factors. First, the introduction of medications or a better compliance to the current treatment as well as to the psychotherapeutic interventions may have positively impacted the participants’ mental health state. Secondly, ICMA may have broken the vicious circle in which this population is often trapped. In fact, severe substance use disorders are associated with social dropout and inappropriate medical care. In these conditions, participants’ needs are misunderstood. Help is often provided only during crisis periods and mainly through compulsion (involuntary admission or guardianship) [32, 33]. The use of coercion can lead to increased anxiety, loss of self-esteem and mood disorders, with a consequent intensification of alcohol and other illicit drug consumption [34]. Focusing more attentively on participants’ primary needs, ICMA offered them a new perspective on their situation. The rapid changes in the participants’ social context may have enhanced their sense of empowerment and self-confidence and given them hope, reducing the rate and frequency of alcohol and other illicit drug use. Conversely, the reduction of substance intake may have decreased cognitive problems and mood disorders, reduced behavioural problems such as self-injury, eased participants’ relations with others and prevented physical complications. This may have positively influenced the number of ED visits.

While in need for further experimentation on a larger sample, the intervention seemed to positively influence the rates of homelessness and victimisation in this highly vulnerable population during the follow-up period. This former finding confirmed the positive impact of ACT and CTI in improving housing stability and reducing the rate of homelessness among people with severe mental and substance use disorders [11]. The latter finding pointed at the ICMA as an excellent approach to reduce victimisation among this highly vulnerable population which gathers several risk factors such as severe mental and substance use disorders, homelessness, and absence of contact with psychiatric services [35, 36].

### Strengths and limitations

Regarding the strengths, this was a prospective exploratory study. Despite the difficult-to-engage target population, none of the participants refused to take part in the research project.

Two main methodological limitations were the absence of a control group and the modest sample size. The intervention was tested on a local level in Lausanne, an urban area of 265′000 inhabitants in the French-speaking part of Switzerland. Generalizability of the results may thus be restricted. Besides, some measures were self-reported while others were rated by the case managers.

## Conclusion

Despite the limited scale of this observational project, the results are promising, highlighting the efficacy of ICMA methodology to improve engagement with care and the psychosocial situation of this hard-to-reach population, with consequent direct impact on substance problems. Further research is needed to confirm the programme effectiveness. However, the high-risk profile of these participants and the dangers related to the lack of an adapted care could represent an ethical and actual obstacle to the assessment of this issue in a randomised controlled trial setting.

The ICMA does not replace other addiction treatment services. Its aim is to locate and design an alternative way to promote participants’ engagement with standard care and to offer people with severe mental and substance use disorders new opportunities, a better control on their lives and an improved well-being.

## Abbreviations

ED: Emergency Department
ICM: Intensive Case Management
ICMA: Intensive Case Management for Addiction
